# Systematic review of economic evaluations for paediatric pulmonary diseases

**DOI:** 10.1186/s12962-023-00423-1

**Published:** 2023-04-30

**Authors:** Mutsawashe Chitando, Susan Cleary, Lucy Cunnama

**Affiliations:** Health Economics Unit and Division, School of Public Health and Family Medicine, Faculty of Health Sciences, University of Cape Town, Anzio Road, Observatory, Cape Town, 7925 South Africa

**Keywords:** Paediatric, Pulmonary disease, Economic evaluation, Systematic review

## Abstract

**Background:**

Paediatric pulmonary diseases are the leading causes of mortality amongst children under five globally. Economic evaluations (EEs) seek to guide decision-makers on which health care interventions to adopt to reduce the paediatric pulmonary disease burden. This study aims to systematically review economic evaluations on different aspects of the inpatient management of paediatric pulmonary diseases globally.

**Methods:**

We systematically reviewed EEs published between 2010 and 2020, with a subsequent search conducted for 2020–2022. We searched PubMed, Web of Science, MEDLINE, Paediatric Economic Database Evaluation (PEDE) and the Cochrane library. We extracted data items guided by the Consolidated Health Economic Evaluation Reporting Standards (CHEERS) checklist. We collected qualitative and quantitative data which we analysed in Microsoft Excel and R software.

**Results:**

Twenty-two articles met the inclusion criteria. Six of the articles were cost-effectiveness analyses, six cost-utility analyses, two cost-minimisation analyses and eight cost analyses. Twelve articles were from high-income countries (HICs) and ten were from low- and middle-income countries (LMICs). Eight articles focused on asthma, eleven on pneumonia, two on asthma and pneumonia, and one on tuberculosis.

**Conclusion:**

Conducting more EEs for paediatric pulmonary diseases in LMICs could allow for more evidence-based decision-making to improve paediatric health outcomes.

## Introduction

Paediatric pulmonary diseases are the leading causes of morbidity and mortality amongst children under the age of five, especially in low- and middle-income countries (LMICs) [1]. According to a report by the World Health Organisation [2], pneumonia is the largest infectious cause of death in children, contributing 14% of all deaths in children under five years old and claiming the lives of 740 180 children in 2019. Fortunately, most paediatric pulmonary diseases can be prevented and controlled through vaccinations, practising good hygiene, and exercising [3]. In instances where a child has severe disease, hospitalisation is often required with key implications for resource utilisation [3]. To manage this burden, decision-makers utilise a variety of planning and budgeting tools to help inform their decisions on resource allocation.

Over the years, economic evaluations (EEs) have increased in availability and have gained more acceptance in priority setting [4]. EEs are an important component of health technology assessment and provide evidence regarding which health care intervention to adopt by comparing the costs and consequences of competing alternatives [4]. Whilst there is a steady increase in the number of EEs conducted for paediatric conditions, there is still a dearth of studies for EEs conducted in hospital settings for paediatric pulmonary diseases [5]. To bridge this gap, this study systematically reviews EEs focusing on inpatient management of paediatric pulmonary diseases conducted globally from 2010 to 2020. In addition, the literature between 2020 and 2022 was assessed. The objectives are to provide a qualitative and quantitative description of existing literature on EEs for inpatient treatment of paediatric pulmonary diseases; categorise the methodologies used for the different EEs; describe the health care and geographical settings of the articles included; and describe the types of diseases and the different interventions that were evaluated.

## Methods

### Search strategy

We conducted a systematic review of EEs focusing on alternative approaches for inpatient management of paediatric pulmonary diseases within five electronic databases: PubMed, Web of Science, MEDLINE, Paediatric Economic Database Evaluation (PEDE), and the Cochrane library. We made use of keyword searches, MeSH terms, truncation, and Boolean operators. We had three search categories, namely: paediatrics, pulmonary disease, and EEs. We also set parameters for the year of publication to include 2010–2020. An updated search was also conducted for 2020–2022 to identify any additional articles.

#### Inclusion criteria

We included full EEs cost-effectiveness analysis (CEA), cost-utility analysis (CUA), cost–benefit analysis (CBA), cost-minimisation analysis (CMA) and partial EEs (cost descriptions, cost analysis and cost of illness studies). We included articles reported in the English language, which were published between the years 2010 and 2020. The EEs included in the review were specific to paediatric pulmonary diseases, comprising inpatients aged from zero to six years old. Our focus was on interventions delivered within the inpatient setting, including alternative medications, diagnostics and screening, medical devices, and additional support such as supplemental oxygen. Countries were included irrespective of income level.

#### Selection process

**Fig. 1:**
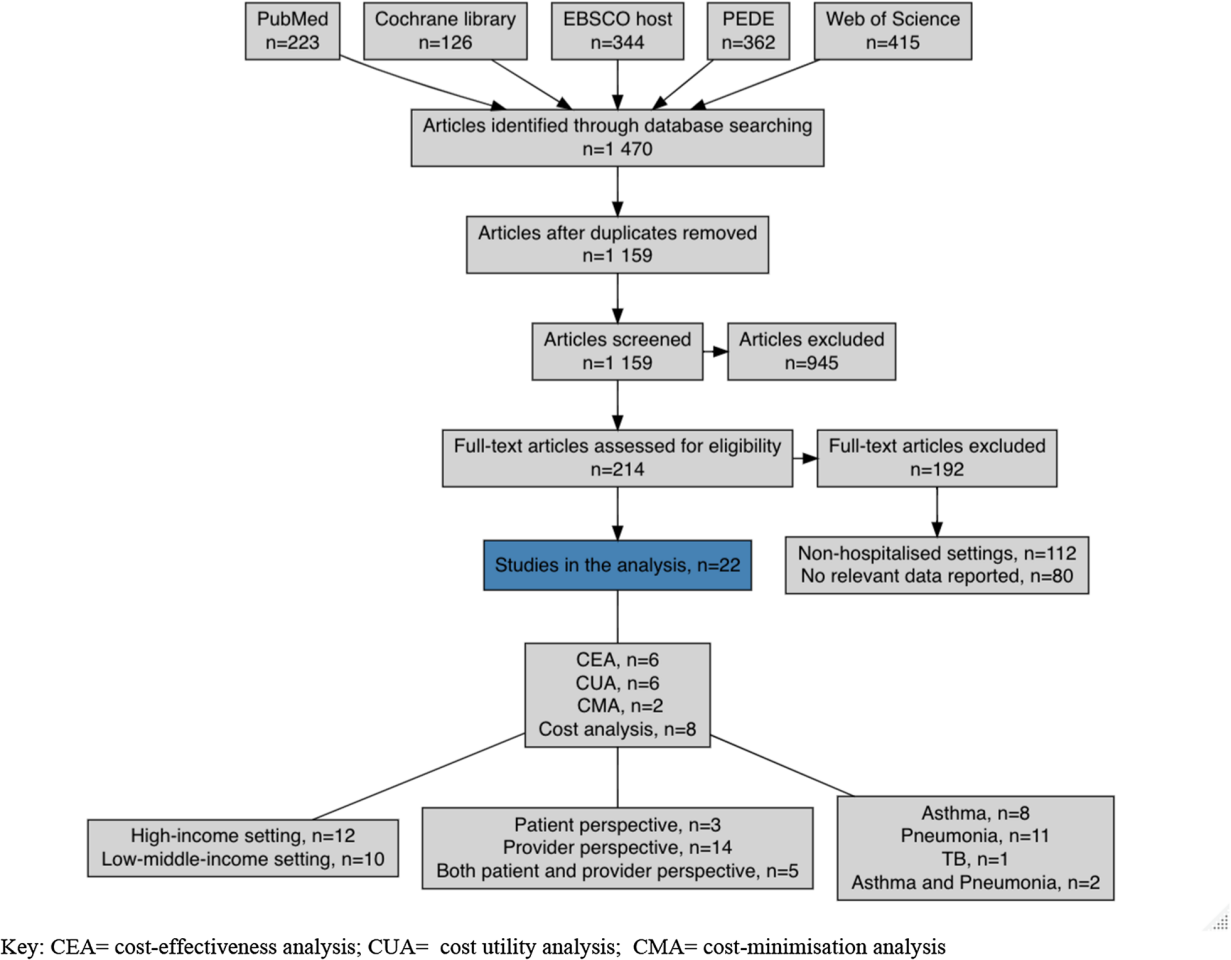
PRISMA diagram for period 2010–2022. *CEA* cost-effectiveness analysis, *CUA* cost utility analysis, *CMA* cost-minimisation analysis

In the first stage of the selection process, we removed duplicates in EndNote X9 Software (Clarivate Analytics). We did this both electronically and manually. The screening of the papers was done in three stages: title screening, abstract screening and full-text screening. These stages are represented diagrammatically in the PRISMA diagram, Fig. 1. The selection process was carried out by one reviewer who was in consultation with a second reviewer for all the steps.

#### Data management

After the selection process, we stored the articles which met the eligibility criteria in a shareable folder in EndNote X9 Software, Clarivate Analytics.

### Data extraction

We developed an extraction tool in Microsoft Excel using the Consolidated Health Economic Evaluation Reporting Standards (CHEERS) checklist as a guide to identify the data items [6]. The CHEERS checklist was used on the premise of its usefulness in ensuring that “*health economic evaluations are identifiable, interpretable, and useful for decision making*” [7]. The extraction tool was pre-tested on five articles for its relevance and appropriateness to the study before use. The variables extracted related to: author, year, title, journal name, funder, study perspective, duration of the study, setting, intervention and comparator, currency reported, type of EE, discounting, sensitivity analysis, informed consent, unit costs, outcome measures and incremental cost-effectiveness ratios (ICERs).

### Data synthesis and analysis

**Fig. 2:**
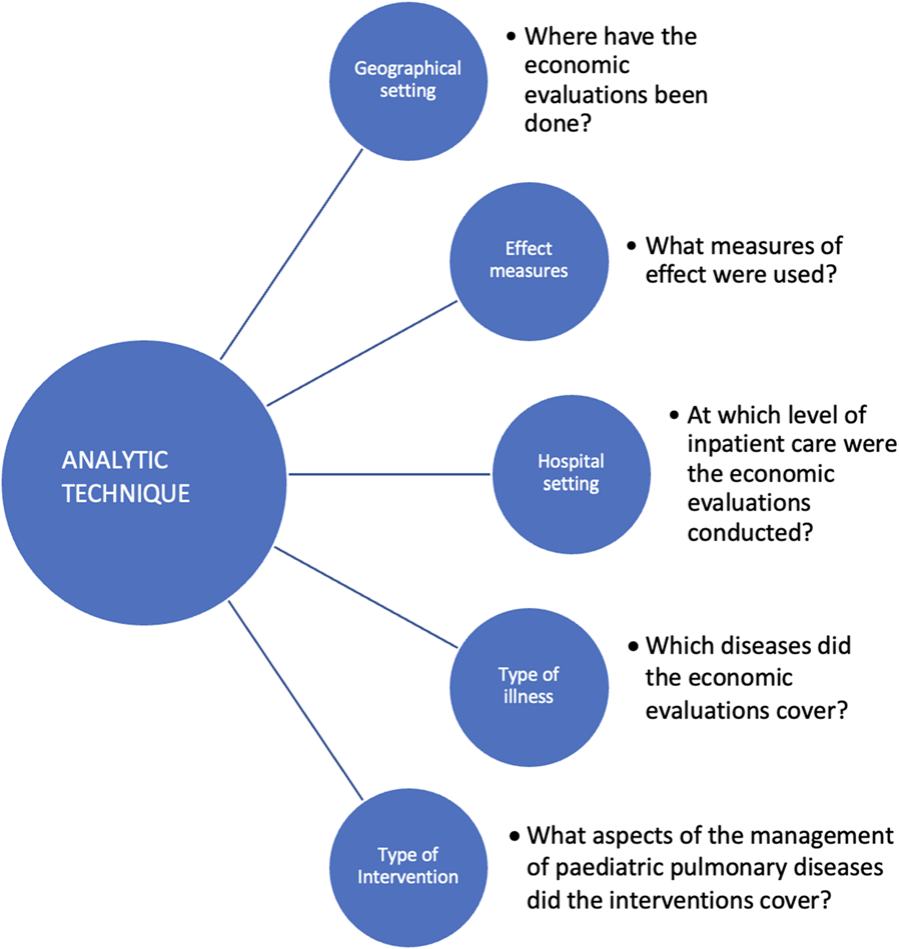
Data analysis framework

We adopted a convergent mixed-methods approach [8], combining both qualitative and quantitative data. For the qualitative assessment, we used a data analysis framework designed during the protocol development stage (Fig. 2). The framework allowed for the comparison between the type of economic evaluation (outcome variable) and other variables of interest (input variables) by positioning the outcome variable at the centre of analysis. For the quality assessment, we utilised the CHEERS 24-point checklist for assessing the reporting standards of the studies included in the review [6]. 5 studies had a high quality score (75–100%), 12 studies had a moderate quality score (50–74%), and 3 studies had a low quality score (< 50%). The quality assessment provides a rationale for the extent to which decision-makers can use health economic evidence in their decision-making based on the reporting quality.

We conducted our quantitative data analysis in Microsoft Excel and R software (R Project, Vienna, Austria) using the RStudio interface. We analysed the volume of publications, the hospitalisation costs, and ICERS. All costs which were not reported in United States Dollars (USD) were converted to USD using the reported exchange rate for the study year. We inflated the costs to 2019 USD using World Bank Consumer Price Indices [9]

### Study approval

The study is a secondary analysis which did not involve human subjects; however, we obtained ethical approval from the Human Research Ethics Committee (HREC) at the University of Cape Town (UCT), reference number HREC 587/2020.

## Results

### Eligibility screening

Our search retrieved 1470 articles. After duplicates were removed, both manually and electronically, 1159 articles remained. Following the screening by title and by abstract, 945 of the 1159 articles were excluded. We then screened the full text of the remaining 214 articles and 20 articles met the full inclusion criteria (Fig. 1). A description of the characteristics of each study included in the systematic review is found in Table 1.

**Table 1:** Characteristics of the articles in the systematic review

Publication year	Lead author	Perspective	Country/ countries	Disease(s)	Intervention(s)	Comparator(s)
2021	Duke, T [21]	Provider	Papua New Guinea	Pneumonia	Solar powered oxygen system	No system
2021	Huang YM [22]	Provider and Patient	Uganda	Pneumonia	Solar powered oxygen	No oxygen and grid powered oxygen
2020	Kitano, T [23]	Provider and Patient	Japan	Asthma and Pneumonia	mPCR tests	Rapid Antigen Tests
2019	Chen, H. H. [24]	Patient	Ethiopia	Pneumonia	Oral antibiotics	Not reported
2018	von Schoen-Angerer, T. [25]	Provider	Switzerland	Asthma and Pneumonia	Standard hospital care	Complementary treatment
2018	Ceyhan, M. [26]	Provider	Turkey	Pneumonia	In-patient treatment	Not reported
2017	Zhang, S [27]	Provider	Uganda, South Africa, Zambia, Zimbabwe	Pneumonia	2013 WHO guidelines	2005 WHO guidelines
2017	Debes, A. K. [17]	Provider	Uganda	TB	MODS, Expert and Empirical	Standard treatment
2016	Bozzani, F. M. [28]	Provider	Malawi	Pneumonia	PCV 13	Pre-intervention
2015	Razi, C. H [29]	Provider	Turkey	Asthma	Nebulisation	Placebo
2015	Andrews, A. L. [30]	Provider	USA	Pneumonia	Targeted blood cultures	Universal blood cultures
2015	Chu, S. M. [31]	Provider	China	Pneumonia	Ventilator use (2 days)	Ventilator use (1 week)
2015	Floyd, J. [32]	Provider	Uganda	Pneumonia	PO1, PO2	IMCI
2014	Petrou, S. [33]	Provider and patient	UK	Asthma	Nebulisation	Standard treatment
2013	Krebs, S. E. [34]	Provider	USA	Asthma	Nebulisation	Standard treatment
2013	Char, D. S. [35]	Provider	USA	Asthma	Volatile anaesthesia	Supplemental oxygen
2013	Powell, C. [36]	Patient	UK	Asthma	Nebulisation	Placebo
2012	Andrews, A. L. [37]	Provider and Patient	USA	Asthma	Prescribe and dispense ICS	Usual care
2012	Andrews, A. L. [38]	Provider and Patient	USA	Asthma	Oral tablets (prednisone)	Oral tablets (dexamethasone)
2011	Doan, Q. [39]	Provider	Canada	Asthma	Metered-dose inhaler	Nebulisation
2011	Broughton, E. I. [18]	Provider	Nicaragua	Pneumonia	Quality improvement	Pre-intervention
2010	Lorgelly, P. K. [40]	Patient	UK	Pneumonia	Oral antibiotics	Intravenous antibiotics

*ICS* inhaled corticosteroids, *mPCR* multiplex polymerase chain reaction, *PCV* pneumococcal conjugate vaccine, *PO1/PO2* partial pressure of oxygen, *IMCI* integrated management of childhood illnesses, *TB* tuberculosis, *UK* United Kingdom, *USA* United States of America, *WHO* World Health Organisation

### Description of articles

Of the 22 articles included, 12 were from HICs [20, 23, 25, 31, 33–40] and 10 were from LMICs [17, 18, 21, 22, 24, 26–29, 32]. For HICs, most were from a United States of America (USA) context [20, 34, 35, 37, 38]. Amongst all articles, six were CEAs [17, 30, 35, 37–39], six were CUAs [18, 21, 27, 32, 33, 36], two were CMAs [25, 40], and eight were cost analyses [22–24, 26, 28, 29, 31, 34] (see Table 2). Of the EEs included 17/22 were trial-based, and 5/22 [17, 22, 30, 32, 39] were model-based.

**Table 2:** Methodological characteristics

Lead author	Reference year	Type of economic evaluation	Trial or model based	Costing data	Sensitivity Analysis	Informed consent	Outcome measures	Study duration (months)	Discount rate	Hospitalisation costs (USD)	ICERS Δcosts (USD)/Δoutcome
Duke, T [21]	2021	Cost analysis	Trial	Prospective	NR	NR	NA	48	NR	NR	NA
Huang YM [22]	2021	Cost-utility analysis	Model	Prospective	Multi-way	NR	DALY averted	12	3%	NR	140
Kitano, T [23]	2020	Cost analysis	Trial	Prospective	NR	Stated- no informed consent	NA	12	NR	1 421.40	NA
Chen, H. H. [24]	2019	Cost analysis	Trial	Retrospective	One-way	NR	NA	12	NR	47.89	NA
von Schoen-Angerer, T. [25]	2018	Cost minimisation analysis	Trial	Prospective	NR	NR	NR	18	NR	NR	NA
Ceyhan, M. [26]	2018	Cost analysis	Trial	Retrospective	Probabilistic	NR	NR	12	NR	1 945.80	NR
Zhang, S [27]	2017	Cost-utility analysis	Trial	Retrospective	One-way	Stated- no informed consent	DALY averted	12	NR	NR	34.33
Debes, A. K. [17]	2017	Cost-effectiveness analysis	Model	Retrospective	Multi-way	NR	Life years gained	NR	3%	NR	39
Bozzani, F. M. [28]	2016	Cost analysis	Trial	Retrospective	One-way	Stated- informed consent	NR	3	NR	6.42	NR
Razi, C. H [29]	2015	Cost analysis	Trial	Retrospective	NR	Stated- informed consent	NR	28	NR	299.00	NR
Andrews, A. L. [30]	2015	Cost-effectiveness analysis	Model	Prospective	Probabilistic	NR	ED visits averted	12	NR	2 030.40	NR
Chu, S. M. [31]	2015	Cost analysis	Trial	Retrospective	NR	NR	NR	30	NR	NR	NR
Floyd, J. [32]	2015	Cost-utility analysis	Model	Prospective	NR	NR	DALY averted	NR	NR	6.44	11.63
Petrou, S. [33]	2014	Cost utility analysis	Trial	Prospective	NR	NR	QALY gained	28	NR	285.36	337.02
Krebs, S. E. [34]	2013	Cost analysis	Trial	Prospective	NR	NR	NR	12	NR	123.76	NR
Char, D. S. [35]	2013	Cost-effectiveness analysis	Trial	Prospective	NR	Stated- no informed consent	Complications avoided	> 48	NR	NR	NR
Powell, C. [36]	2013	Cost utility analysis	Trial	Prospective	NR	Stated- informed consent	QALY gained	NR	NR	1 549.29	189
Andrews, A. L. [37]	2012	Cost-effectiveness analysis	Trial	Prospective	Two-way	NR	ED visits averted	NR	NR	7 244.64	NR
Andrews, A. L. [38]	2012	Cost-effectiveness analysis	Trial	Prospective	Two-way	NR	ED visits averted	NR	NR	7 244.64	NR
Doan, Q. [39]	2011	Cost-effectiveness analysis	Model	Retrospective	One-way	Stated- no informed consent	ED visits averted	NR	NR	2 857.19	-3 033.31
Broughton, E. I. [18]	2011	Cost utility analysis	Trial	Retrospective	One-way	NR	DALY averted	24	3%	280.17	-396.00
Lorgelly, P. K. [40]	2010	Cost minimisation analysis	Trial	Prospective	One-way	NR	ED visits averted	24	NR	870.46	NR

*NR *no reported, *ED *visits emergency department visits, *NA* not applicable, *Δ* difference in

Using our data analysis framework (Fig. 2), we identified 8 articles on asthma [29, 33–39], eleven on pneumonia [18, 20–22, 24, 26–28, 31, 32, 40], two on both asthma and pneumonia [23, 25] and one on tuberculosis (TB) [17]. Articles which covered asthma were predominantly from HICs (7/8) [33–39], pneumonia articles were fairly evenly distributed between HICs [20, 25, 31, 40] and LMICs [18, 21, 22, 24, 26–28, 32], and the only TB study [17] was from a LMIC. The interventions evaluated included diagnostic tests, operational guidelines, antibiotic use (oral vs intravenous), inhaled corticosteroids and supplementary oxygen.

### Cost data

We extracted hospitalisation costs and ICERs, where relevant, for all articles included in the systematic review. Table 2 summarises the methodological characteristics of the included articles. It shows the author details, reference year, type of economic evaluation, costing data, sensitivity analysis, informed consent, outcome measures, study duration, discount rate, hospitalisation costs (in USD) and ICERs (in USD). 77% (17/22) [18, 23, 24, 26, 28–30, 32–40] of the articles reported hospitalisation costs, and 71% (10/14) of the full EEs reported ICERs [17, 18, 22, 27, 32, 33, 36, 39]. The highest hospitalisation cost reported was USD7 245, the lowest hospitalisation cost was USD6 and the median hospitalisation cost was USD285.

### Methodology

For the methodology, three articles adopted a patient perspective [24, 36, 40], fourteen a provider perspective [17, 18, 20, 21, 25–29, 31, 32, 34, 35, 39] and five a societal (patient and provider) perspective [22, 23, 33, 37, 38]. The costing for 13 of the articles was done prospectively [21–23, 25, 26, 30, 32–35, 37, 38, 40], and the remainder were retrospective [17, 18, 24, 26–29, 31, 39]. We also assessed the reporting of informed consent in the included articles and found that 68% did not state whether they had collected informed consent [17, 18, 21, 22, 24–26, 30–34, 37, 38, 40], 14% reported consent [28, 29, 36] and 18% reported no informed consent process [23, 27, 35, 39]. Some of the reasons for not reporting consent in the reviewed articles included exemption status or waivered informed consent, and then some articles simply did not report whether there was informed consent or not.

With regards to sensitivity analysis, 55% (12/22) [17, 18, 22, 24, 26–28, 30, 37–40] of the articles reported performing a sensitivity analysis and the remaining 45% (10/22) did not. Of those that reported on sensitivity analysis, the most common type of sensitivity analysis was a one-way sensitivity analysis, reported by 42% (5/12) of these articles [18, 27, 28, 39, 40]. Table 3 shows the results of the quality assessment of each study. Only two articles reported discounting [17, 18], and this was at a rate of 3% for both costs and outcomes.

**Table 3:** Quality assessment

Lead author	Reference year	1	2	3	4	5	6	7	8	9	10	11	12	13
Duke, T [21]	2021	Yes	Yes	Yes	Yes	Yes	No	Yes	Yes	No	Yes	No	No	Yes
Huang YM [22]	2021	Yes	Yes	Yes	Yes	Yes	No	Yes	Yes	Yes	Yes	Yes	No	Yes
Kitano, T [23]	2020	No	Yes	Yes	Yes	Yes	Yes	Yes	Yes	No	Yes	No	No	Yes
Chen, H. H. [24]	2019	No	Yes	Yes	Yes	Yes	Yes	Yes	No	No	No	No	No	Yes
von Schoen-Angerer, T. [25]	2018	No	Yes	Yes	Yes	Yes	Yes	Yes	Yes	No	Yes	Yes	No	Yes
Ceyhan, M. [26]	2018	No	Yes	Yes	Yes	Yes	Yes	No	Yes	No	Yes	No	No	Yes
Zhang, S [27]	2017	Yes	Yes	Yes	Yes	Yes	Yes	Yes	Yes	No	Yes	Yes	No	Yes
Debes, A. K. [17]	2017	Yes	Yes	Yes	Yes	Yes	Yes	Yes	Yes	Yes	Yes	Yes	No	Yes
Bozzani, F. M. [28]	2016	No	Yes	Yes	Yes	Yes	Yes	Yes	Yes	No	No	No	No	Yes
Razi, C. H [29]	2015	No	Yes	Yes	Yes	Yes	Yes	Yes	Yes	No	Yes	No	No	Yes
Andrews, A. L. [30]	2015	Yes	Yes	Yes	Yes	Yes	Yes	Yes	Yes	No	Yes	Yes	No	Yes
Chu, S. M. [31]	2015	No	Yes	Yes	Yes	Yes	Yes	Yes	Yes	No	Yes	No	No	Yes
Floyd, J. [32]	2015	No	Yes	Yes	Yes	Yes	Yes	Yes	No	No	No	Yes	No	Yes
Petrou, S. [33]	2014	Yes	Yes	Yes	Yes	Yes	Yes	Yes	Yes	No	Yes	Yes	Yes	Yes
Krebs, S. E. [34]	2013	No	Yes	Yes	Yes	Yes	Yes	Yes	No	No	Yes	No	No	Yes
Char, D. S. [35]	2013	Yes	Yes	Yes	Yes	Yes	Yes	Yes	Yes	Yes	Yes	No	No	Yes
Powell, C. [36]	2013	Yes	Yes	Yes	Yes	Yes	Yes	Yes	Yes	No	Yes	Yes	Yes	Yes
Andrews, A. L. [37]	2012b	Yes	Yes	Yes	Yes	Yes	Yes	Yes	No	No	Yes	No	No	Yes
Andrews, A. L. [38]	2012a	Yes	Yes	Yes	Yes	Yes	Yes	Yes	Yes	No	Yes	No	No	Yes
Doan, Q. [39]	2011	Yes	Yes	No	Yes	Yes	Yes	Yes	Yes	No	Yes	Yes	No	Yes
Broughton, E. I. [18]	2011	Yes	Yes	Yes	No	Yes	Yes	Yes	Yes	Yes	Yes	No	Yes	Yes
Lorgelly, P. K. [40]	2010	Yes	Yes	Yes	Yes	Yes	Yes	Yes	Yes	No	Yes	Yes	No	Yes

Lead author	Reference year	14	15	16	17	18	19	20	21	22	23	24	% of reporting standards met
Duke, T [21]	2021	Yes	No	No	Yes	Yes	Yes	No	No	Yes	Yes	Yes	66.7
Huang YM [22]	2021	Yes	Yes	Yes	Yes	Yes	Yes	Yes	No	Yes	Yes	Yes	87.5
Kitano, T [23]	2020	No	No	No	No	No	Yes	No	No	Yes	Yes	No	50
Chen, H. H. [24]	2019	Yes	No	No	Yes	No	No	Yes	No	Yes	No	No	45.8
von Schoen-Angerer, T. [25]	2018	No	No	Yes	No	No	No	No	No	Yes	No	No	50
Ceyhan, M. [26]	2018	No	No	No	No	Yes	Yes	No	No	Yes	Yes	No	50
Zhang, S [27]	2017	Yes	Yes	No	Yes	Yes	Yes	Yes	No	Yes	Yes	Yes	79.2
Debes, A. K. [17]	2017	No	No	Yes	Yes	Yes	Yes	Yes	Yes	Yes	Yes	No	83.3
Bozzani, F. M. [28]	2016	No	No	No	No	No	Yes	No	No	Yes	Yes	No	45.8
Razi, C. H [29]	2015	No	No	No	No	No	No	No	No	Yes	No	No	41.2
Andrews, A. L. [30]	2015	Yes	Yes	Yes	Yes	Yes	No	No	No	Yes	No	No	70.8
Chu, S. M. [31]	2015	No	No	No	Yes	Yes	No	No	No	Yes	No	No	50
Floyd, J. [32]	2015	No	Yes	No	Yes	Yes	Yes	Yes	No	Yes	Yes	No	62.5
Petrou, S. [33]	2014	Yes	No	No	Yes	Yes	Yes	Yes	No	Yes	Yes	Yes	83.3
Krebs, S. E. [34]	2013	Yes	No	No	No	No	Yes	No	No	Yes	No	No	45.8
Char, D. S. [35]	2013	Yes	No	No	No	No	No	No	No	Yes	No	No	54.2
Powell, C. [36]	2013	Yes	Yes	Yes	Yes	No	Yes	Yes	No	Yes	Yes	No	83.3
Andrews, A. L. [37]	2012b	Yes	Yes	Yes	Yes	Yes	Yes	No	Yes	Yes	No	No	70.8
Andrews, A. L. [38]	2012a	Yes	Yes	Yes	Yes	No	No	Yes	No	Yes	No	No	66.7
Doan, Q. [39]	2011	No	Yes	No	Yes	Yes	Yes	Yes	No	Yes	No	No	66.7
Broughton, E. I. [18]	2011	Yes	Yes	No	Yes	Yes	Yes	Yes	No	Yes	No	No	75
Lorgelly, P. K. [40]	2010	Yes	No	No	Yes	Yes	No	Yes	No	Yes	Yes	No	70.8

Checklist: 1. Title; 2. Abstract; 3. Introduction 4. Target Population; 5. Setting and Location; 6. Study Perspective; 7. Comparators; 8. Time Horizon; 9. Discount Rate; 10. Choice of health outcomes; 11a. Measurement of effectiveness (single study-based estimates); 11b. Measurement of effectiveness (synthesis-based estimates); 12. Measurement of preference-based outcomes; 13a. Estimating Resources and Costs (single study-based economic evaluation); 13b. Estimating Resources and Costs (model-based economic evaluation); 14. Currency, Price, Conversion; 15. Model Choice; 16. Assumptions; 17. Analytical Methods; 18. Study Parameters; 19. Incremental Costs and Outcomes; 20a. Characterizing Uncertainty (single study based economic evaluation); 20b. Characterizing Uncertainty (model-based economic evaluation); 21. Heterogeneity; 22. Study Findings; 23. Funding; 24. Conflicts of Interest

*Yes* reported in full or partially, *No* not reported or not clear. Quality ranking Yes = 1; No = 0

### Outcome measures

We also used our data analysis framework to identify the outcome measures, which were reported in the articles as natural units, quality adjusted life years (QALYs) and disability adjusted life years (DALYs). The natural units were emergency department (ED) visits averted (5) [30, 37–40], life-years gained (1) [17], and complications avoided (1) [35] Two articles reported QALYs gained [33, 36] and four reported DALYs averted [18, 22, 27, 32].

## Discussion

The importance of EEs being readily available to inform health care priority setting must be underscored. In this regard, systematic reviews such as this can synthesise large amounts of economic evaluation data and make these more accessible [10]. The findings from our systematic review were indicative of more EEs being conducted in HICs compared to LMICs [5]; given the need for context specific findings, this points to a key gap in the literature regarding inpatient care for pulmonary diseases in children in LMICs. These findings were consistent with those from a study by Ungar and Zur [11], where they noted that whilst there was an increase in the number of EEs globally, there were more EEs reported in HICs than there were in LMICs. An explanation for this could be limited analytical resources and research funding to conduct the EEs in these setting [12].

Our systematic review identified both partial EEs (cost-analysis) and full EEs (CEAs, CUA, CMAs). Cost analysis is the most basic form of (partial) EE as it assesses only the costs of the intervention and provides no information on the outcomes [13]. The results of partial EEs are fairly comprehendible for decision-makers, which could explain why they were more of them than other types of EEs. Some policy makers lack sufficient knowledge in interpreting the findings of full EEs, and consequently may be hesitant about using them to inform policy. We also identified that not many studies met the standards of high-quality reporting, thereby limiting the uptake of these results by policy makers on priority setting.

We also highlight some notable differences in the geographical distribution of diseases for which the EEs were conducted. This could be attributed to asthma being a disease of affluence [14]. In the case of pneumonia, it disproportionately affects less-affluent countries [15], which could explain the wider availability of EEs for pneumonia inpatient interventions in LMICs.

Interestingly, we only found one TB study for the zero to six age group, yet TB incidence is high in LMICs [15]. A possible explanation for this could be that TB is largely managed on an outpatient basis, while our systematic review focused on inpatient settings. Another alternative is that the economics of TB treatment in children has not been well researched [16].

We were also interested in understanding the different perspectives adopted in the articles. There were more articles which adopted the provider perspective than the patient perspective. A possible explanation is one similar to the adoption of full vs partial EEs where the resources for societal costing might not be available.

Our review also summarised the methodological approaches that were employed in the different articles included in the review. From our findings, not all eligible studies reported discounting their costs and outcomes. The studies which discounted their costs and outcomes discounted at a 3% discount rate [17, 18] which is on a par with the 0–5% standard in economic evaluation literature [19]. Whilst both QALYs gained and DALYs averted were used as outcome measures, DALYs averted were more commonly used. We could attribute this in part to difficulties in measuring and valuing utilities in children for QALYs [11]. Ungar et.al (2015), affirm that children are not just little humans, and therefore there is need to develop tools that are specific to them when measuring their quality of life.

### Study strengths and limitations

It is worth highlighting the study strengths and limitations. This systematic review is unique in that it focused on the different types of economic evaluations conducted for paediatric pulmonary diseases in a global context. Unfortunately it only considered EEs conducted in a hospital setting or that assessed inpatients. Therefore, our findings would not be generalizable to other service delivery platforms. The inclusion of only published literature and not grey literature is another limitation. Additionally, screening for eligibility and data extraction was not done by two independent reviewers.

There was also a missed opportunity to analyse the trends in methodological approaches over a longer duration as the review only included published literature between 2010 and 2022 due to practicality.

## Conclusion

The study set out to summarise EEs that have been conducted for paediatric pulmonary diseases globally. There were fewer EEs conducted in LMICs than in HICs, yet children from LMICs are disproportionately affected by pulmonary diseases. Conducting more EEs, of good quality for paediatric pulmonary diseases in LMICs could allow for more evidence-based decision-making to improve paediatric health outcomes.

## Data Availability

Not applicable.

## Search string

The search string in April 2020 across all the databases was:

**Table:**

["paediatric" OR "paediatrics" OR "pediatric" OR "child" OR "children" OR "infant" OR "infants" OR "neonate" OR "neonates"] AND ["pneumonia" OR "asthma" OR "pulmonary TB" OR "bronchiolitis" OR "bronchitis" OR "respiratory infections" OR "paediatric disease" OR "pediatric disease"] AND; ["economic evaluation" OR "economic eval*" OR "economic*" OR "costs" OR "cost-effectiveness" OR "cost-utility analysis" OR "effectiveness" OR "cost–benefit" OR "cost*" OR "cost benefit" OR "cost effectiveness" OR "cost utility analysis" OR "CEA" OR "CUA" or "CBA"]	

The authors can provide access to the search results upon request.
